# Thrombotic Thrombocytopenic Purpura Treated Successfully in a COVID-19 Patient Having a Computed Tomography Severity Score of 22/25

**DOI:** 10.7759/cureus.33097

**Published:** 2022-12-29

**Authors:** Twinkle Pawar, Keyur Saboo, Pallavi Yelne, Shilpa A Gaidhane, Sunil Kumar, Sourya Acharya

**Affiliations:** 1 Medicine, Jawaharlal Nehru Medical College, Datta Meghe Institute of Medical Sciences, Wardha, IND; 2 Internal Medicine, Jawaharlal Nehru Medical College, Datta Meghe Institute of Medical Sciences, Wardha, IND; 3 Epidemiology and Public Health, Jawaharlal Nehru Medical College, Datta Meghe Institute of Medical Sciences, Wardha, IND

**Keywords:** plasmic score, plasmapheresis, ischemic stroke, thrombotic thrombocytopenic purpura, covid-19

## Abstract

We present a case of a 50-year-old female who came to us with chief complaints of breathlessness, general weakness, and cough. She tested positive for coronavirus disease 2019 (COVID-19) on testing with Reverse Transcription Polymerase Chain Reaction (RT-PCR). She had high resolution computed tomography (HRCT) thorax score of 22/25. On investigation, she had thrombocytopenia with schistocytosis on the peripheral smear and evidence of acute kidney injury. She was diagnosed with thrombotic thrombocytopenic purpura (TTP) and was treated with oral prednisone, plasma exchange, and remdesivir. There was an improvement in clinical as well as biochemical parameters such as lactate dehydrogenase, haemoglobin, and platelet counts. This case report highlights TTP that may be a serious complication in COVID-19 patients, especially with a CT severity score of 22/25. Early diagnosis and intervention can lead to a positive outcome.

## Introduction

Thrombotic Thrombocytopenic Purpura (TTP) is a type of microangiopathic haemolytic anaemia characterised by thrombocytopenia, fever, haemolytic anaemia, renal dysfunction, and neurological dysfunction. TTP might be congenital or acquired, and a decrease or absence of the enzyme ADAMTS13 (a disintegrin-like metalloproteinase domain with thrombospondin type 1 motif member 13) is a responsible factor for the causation of TTP [1]. Neurological symptoms are only present in one-third of the population, and usually, acute kidney injury (AKI) is not severe. Unfortunately, there are no specific symptoms that can define TTP, but patients can present with fatigue, gastrointestinal symptoms of nausea and diarrhoea, etc. Low levels of ADAMTS13 result in microthrombi formation that leads to end-organ ischemia and damage. Initial data show that haematological complications are the result of the state of hypercoagulability caused by the immune response to infection by severe acute respiratory syndrome coronavirus 2 (SARS-COV-2) [1]. Autoimmune diseases also have been reported in association with COVID-19 infection. In the literature, we find few reports of COVID-19 associated with autoimmune TTP in pregnancy and paediatric patients. They also revealed improvement in clinical and biochemical parameters on early treatment with plasmapheresis, corticosteroids, and antiviral drugs [2-5]. Here we present a case of a 50-year-old female with COVID-19 associated with TTP with a CT severity score of 22/25 improved very well with corticosteroids, plasmapheresis, and remdesivir.

## Case presentation

A 50-year-old female presented with complaints of breathlessness, general weakness, and cough for two days. She denied any history of fever, chest pain, palpitations, abdominal pain, vomiting, or loose stools. The patient denied a past medical history of diabetes mellitus, hypertension, bronchial asthma, and chronic obstructive pulmonary disease. On physical examination she was afebrile, her pulse was 110/min, blood pressure was 160/100 mmHg, respiratory rate was 26 breaths per minute, and saturation was 96% on oxygen at 2 liters/min. She had bilateral coarse crackles present all over the chest. The rest of the systemic examination was within normal limits.

**Figure 1:**
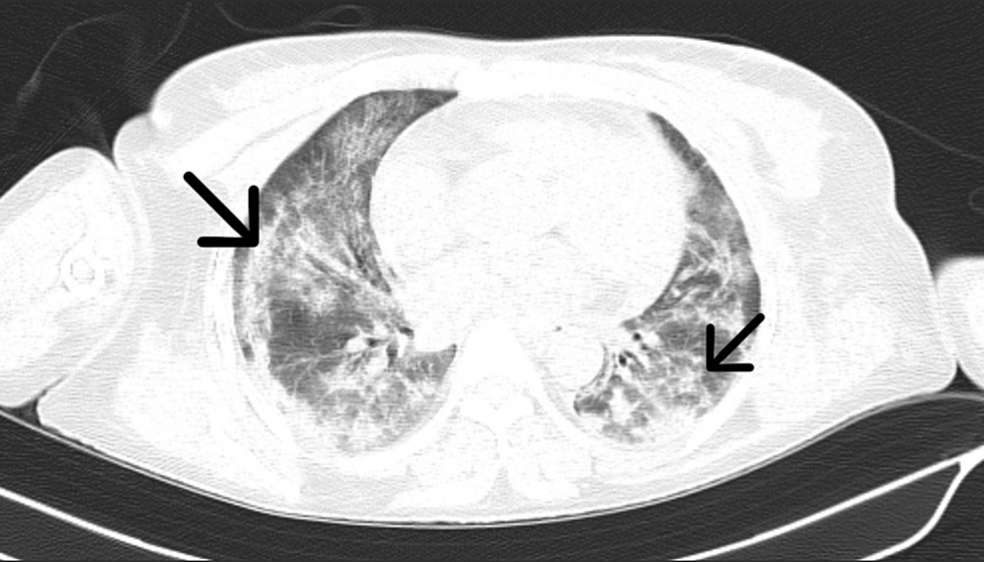
High-resolution computed tomography (HRCT) of the thorax (axial view) Black arrows show ground glass opacities in the lungs

Her coronavirus disease 2019 (COVID-19) polymerase chain reaction (PCR) test was positive. High-resolution computed tomography (HRCT) chest was suggestive of multiple ill-defined patchy ground glass opacities with septal thickening and consolidation in bilateral lung fields with a CT severity score of 22/25 (severe) (Figure 1). Initial laboratory investigations are shown in Table 1. 

**Table 1:** Laboratory investigation values MCV: Mean corpuscular volume

Parameters	Values	Normal range
Complete blood count	Hemoglobin-10.4 gm/dl,	11.6-16.6 gm/dl
	Platelet-50,000/ml	150,000-450,000/ml
	Whole blood count -21,000 cell/ccmm	4000-10,000 cell/ccmm
	MCV- 82.2	80-100
Erythrocyte sedimentation rate	48 mm/hr	0-29 mm/hr
C-reactive protein	18.4mg/dl	<1.0 mg/dl
Ferritin	470 ng/ml	11.1-264 ng/ml
Lactate dehydrogenase	876 mg/dl	100-159 mg/dl
Uric acid	2.8 mg/dl	2.5-6.5 mg/dl
Vitamin B12	159 pg/ml	239-931 pg/ml
Vitamin D	19.5 ng/ml	Deficient- <20 ng/ml Insufficient- 20-<30 ng/ml Sufficient- 20-100 ng/ml
International normalised ratio	1.03	<1.1
Activated partial thromboplastin clotting time	30.9 sec	25-35 sec
Serum creatinine	1.0 mg/dl	0.6-1.25 mg/dl
Reticulocyte count	2.7%	0.5-2.5%

**Figure 2:**
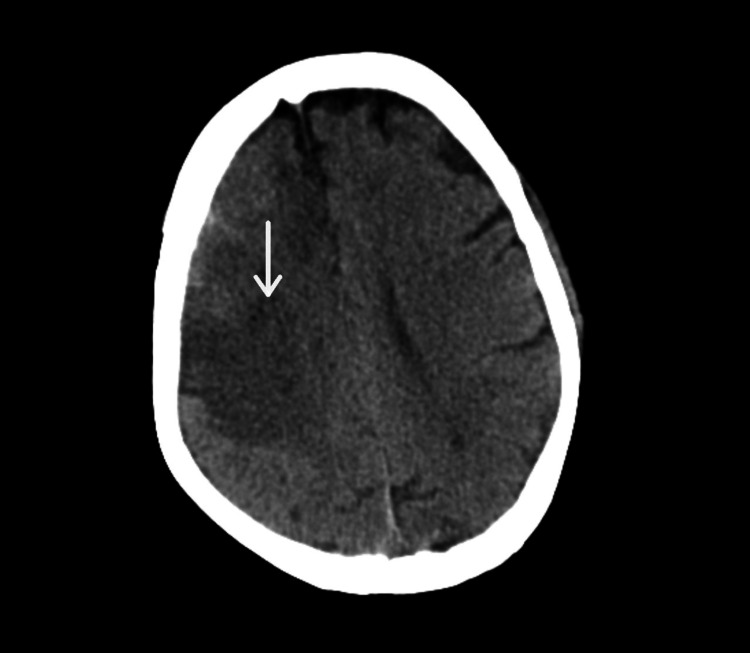
Computed tomography (CT) of the brain Large infarct in right Fronto-Parieto-Temporal lobes (white arrow)

**Figure 3:**
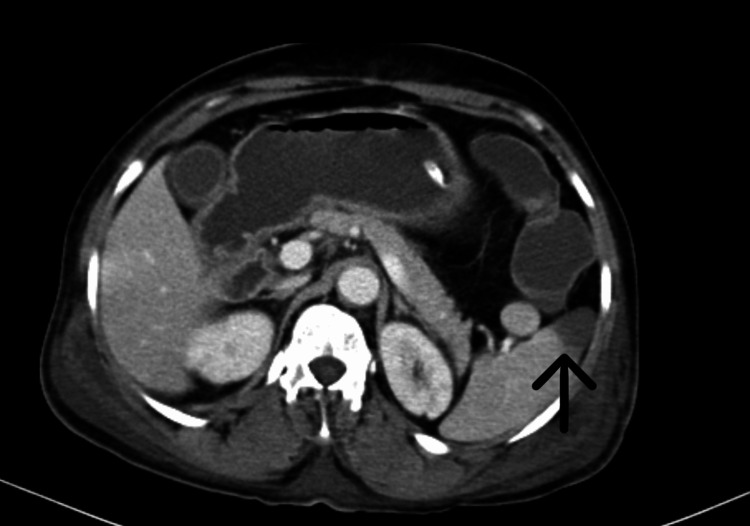
Contrast-enhanced computed tomography (CECT) of the abdomen (axial view) The black arrow shows splenic infarct

**Figure 4:**
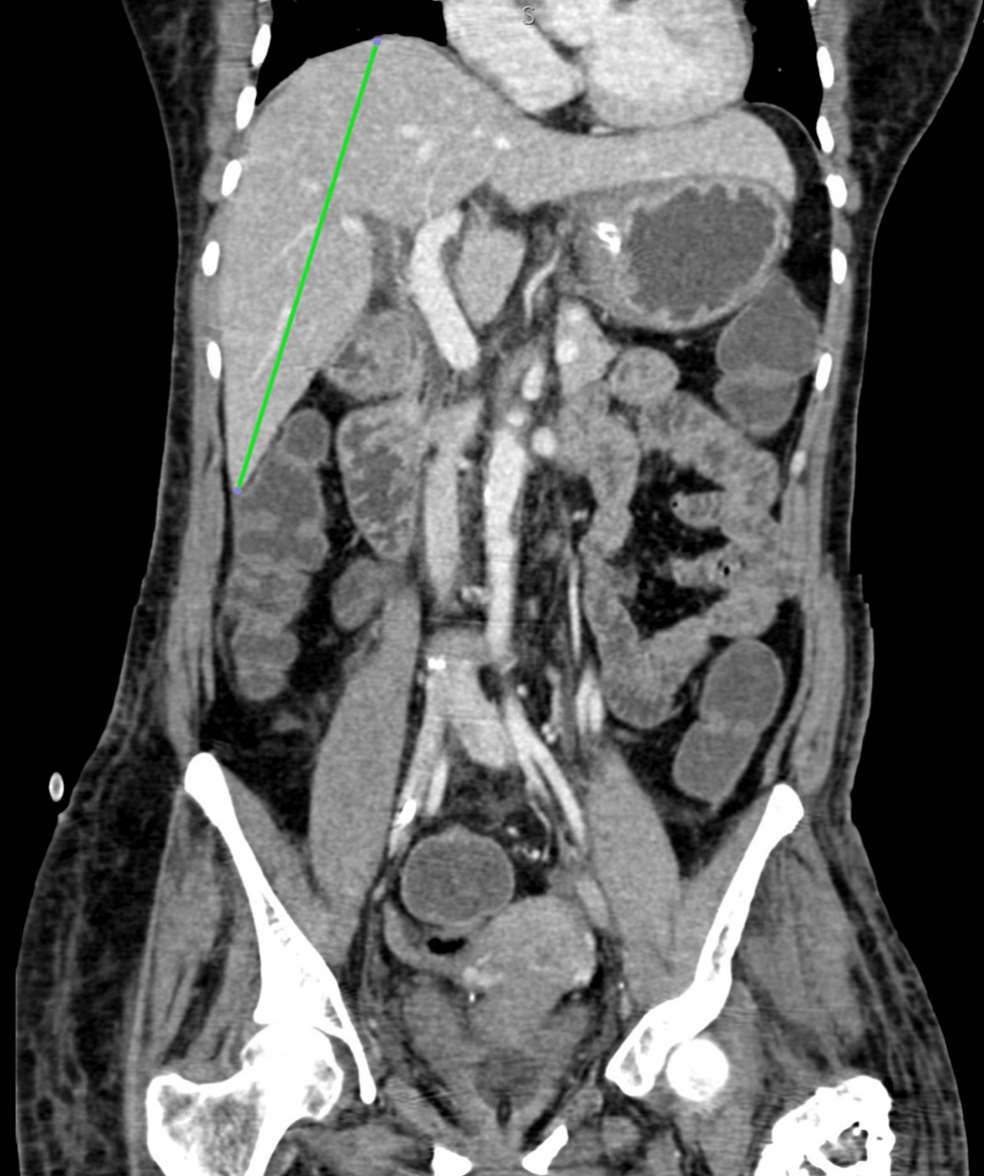
Contrast-enhanced computed tomography (CECT) of the abdomen (sagittal view) The green line shows hepatomegaly

**Figure 5:**
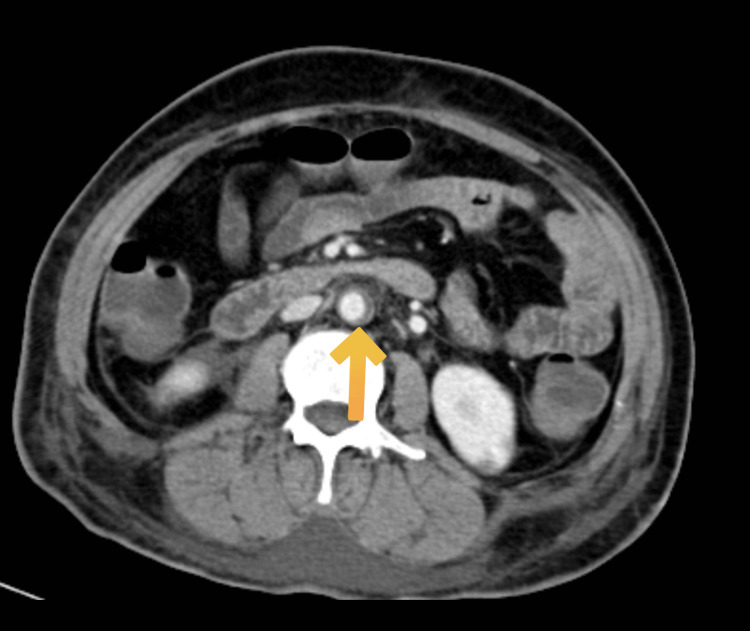
CECT abdomen (axial view) CECT: Contrast-enhanced computed tomography Eccentric partial atherosclerotic thrombus in the abdominal aorta (yellow arrow)

She was treated with remdesivir injection, 200 mg, on day one followed by 100 mg once a day for the next five days, low molecular weight heparin 0.6 mg once a day, injection dexamethasone 6 mg once a day, and other supportive management for six days. She was also treated with piperacillin injection and tazobactam 4.45 gm thrice a day for six days and levofloxacin 750 mg once a day for six days in view of raised white cell counts. On the sixth day of her hospital stay, she had sudden onset left-sided upper and lower limb weakness along with abdominal pain. On neurological examination, the patient was drowsy, power was 0/5 in the left upper and lower limb, and deep tendon reflexes were brisk on the left side with the left extensor plantar. The abdominal examination revealed tenderness in the upper abdomen. In view of these symptoms CT brain and contrast-enhanced computed tomography (CECT) abdomen were done that were suggestive of a large infarct in right Fronto-Parieto-Temporal lobes (Figure 2), splenic infarct (Figure 3) in the mid pole region with spleniculi at the splenic hilum, hepatomegaly (Figure 4) and eccentric partial atherosclerotic thrombus in the abdominal aorta (Figure 5). 

On the same day, her blood investigations revealed profound anaemia with thrombocytopenia. Haemoglobin was 6.3 gm/dl, platelet 0.41 cell/ccmm, and reticulocyte count 2.7%. serum lactate dehydrogenase was done to confirm intravascular hemolysis for the diagnosis of TTP.

**Figure 6:**
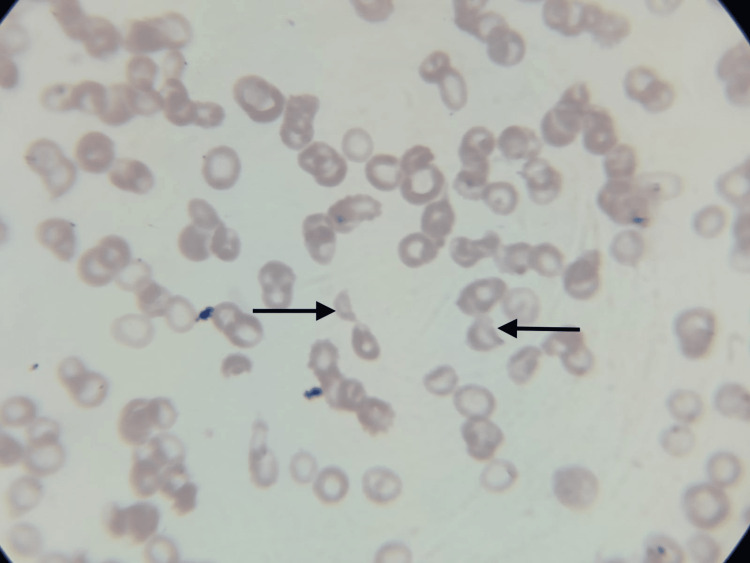
Peripheral smear showing schistocytes

The presence of anaemia, thrombocytopenia, schistocytes on a peripheral smear (Figure 6), indirect hyperbilirubinemia, raised lactate dehydrogenase (LDH) level with encephalopathy and splenomegaly and splenic infarct on CECT abdomen was pointing towards the diagnosis of thrombotic thrombocytopenic purpura.

The patient's PLASMIC score was calculated on the basis of platelet count, serum creatinine, international normalised ratio, mean corpuscular volume, presence of hemolysis, absence of active cancer, and no prior stem cell or organ transplant as shown in Table 2 [6]; in our patient, the score was 6. indicating a high probability of severe ADAMTS13 enzyme deficiency. ADAMTS13 enzyme level was measured to be <10%. So, plasmapheresis along with the management of COVID-19 was started and continued. Sixteen sessions of plasmapheresis were completed with fresh frozen plasma after which an improvement in GCS (Glasgow coma score) was seen. There was an improvement in haemoglobin and platelet counts as well as a concurrent decrease in WBC count, ferritin, C-reactive protein (CRP), and D-dimer, implying that COVID-19's inflammatory process plays a role in TTP pathogenesis. After plasmapheresis treatment completion, the patient was discharged in good general condition.

**Table 2:** PLASMIC score Retic count: Reticulocyte count

Parameter	Result	Score
Platelet count	<30,000	1
Creatinine	<2.0	1
International normalized ratio	<1.5	1
Mean corpuscular volumn	<90	1
Presence of haemolysis	Either- retic count >2.5/ undetectable haptoglobin/indirect billirubin >2 mg/dl	1
Absence of active cancer		1
No prior stem cell or organ transplant		1

## Discussion

COVID-19, along with its pulmonary manifestations, has been known to involve various other systems like gastrointestinal, immunological, cardiovascular, neurological, and hematological systems. Activating coagulation factors, lowering vitamin K levels, and acute sickness that compromises the endothelium barrier are a few of the ways that might increase thrombogenicity and cause problems. Viral infections are a known cause of secondary or immunological TTP, with postulated mechanisms including both direct endothelium damage and the production of ADAMTS13 autoantibodies. However, a causal relationship between the two has not yet been determined. Another theory for the hypercoagulable condition caused by COVID-19 is that it causes endothelial cells to suffer direct cytopathic damage as a result of entrance through angiotensin-converting enzyme 2 (ACE-2) receptors. Additionally, ADAMTS13 levels fall as a result of COVID-19 inflammation, according to small-scale research. A systematic review was done on COVID-19 and its association with TTP, which were unrelated to its severity [7]. Unlike earlier investigations, we found TTP in people who had no history of haematological illness. Previous cases of new-onset TTP during or post COVID-19 infection have been reported. Early diagnosis of TTP is important in the case of COVID-19 as it is a hypercoagulable condition, which increases the risk of thrombus formation in the artery, resulting in acute ischemic stroke [8]. Along with COVID-19 management, the patient was started on plasmapheresis and steroids resulting in an improvement in the platelet, haemoglobin, and LDH levels. Other causes were ruled out, and her COVID-19 swab test came back positive, indicating that this was the most likely reason. After 16 days of COVID-19 care with plasmapheresis, her COVID-19 swab test came negative. Early detection, quarantine, and treatment led to a favourable outcome.

## Conclusions

The pathophysiology and management of TTP in COVID-19 especially with multi-organ involvement can be challenging and need a multidisciplinary approach. This was the case of a COVID-19 patient with TTP, with no prior history of any haematological illness and development of ischemic stroke during hospitalisation. This case highlights the fact that TTP may occur with a severe form of COVID-19 infection. Clinicians should be aware of this association for prompt recognition and timely treatment as early diagnosis and management led to a good outcome.
